# *In Situ* SEM Nanomanipulation and Nanomechanical/Electrical Characterization

**DOI:** 10.1155/2017/8016571

**Published:** 2017-11-05

**Authors:** Yang Lu, Yajing Shen, Xinyu Liu, Mohd Ridzuan Bin Ahmad, Yan Chen

**Affiliations:** ^1^City University of Hong Kong, Kowloon, Hong Kong; ^2^University of Toronto, Toronto, ON, Canada; ^3^Universiti Teknologi Malaysia, Johor, Malaysia; ^4^Arizona State University, Mesa, AZ, USA

In the past decades, in situ scanning electron microscopy (SEM) has become a powerful technique for the experimental study of nanomaterials, since it can provide unprecedented details for individual nanostructures upon mechanical and electrical stimulus, uncovering the fundamental deformation and failure mechanisms for their device applications, such as nanoelectronics, solar cells, and sensors. This special issue aims at exhibiting the latest research achievements, findings, and ideas in the field of in situ SEM nanomanipulation, nanomechanical/electrical characterization, and nanoassembly. In order to give clear introduction and guideline to researchers new to this field, C. Jiang et al. firstly offered an overview about some recent progresses from the literature. They have classified the recent advances of in situ SEM mechanical characterization techniques into tensile, compression, indentation, and bending tests. The state-of-the-art electromechanical coupling analysis was also discussed. Finally, the history of micro/nanomanipulation techniques was also presented, including the critical challenges for the development and design of robust in situ SEM characterization.

Among the original researches reported in this special issue, N. Yu et al. characterized both the resistance and the force at a CNT/Au side-contact interface inside SEM by nanomanipulation technique before and after electron beam induced deposition (EBID). Their robotic system could be expanded to investigate the contact between CNTs and other metals and to fabricate nanodevices such as CNT-FETs in combination with EBID. On the other hand, C. Jiang et al. investigated the torsion fracture behavior of La_50_Al_30_Ni_20_ MG microwire under in situ SEM and compared the fracture surface with tensile loading test based on a self-developed micro robotic mechanical testing system. The fracture mechanism of the microwire under torsional loading was also proposed. They believed this micro robotic system could also be used in many other applications in the future, for example, microassembly of nanoelectronic devices and nanomanufacturing of hierarchical low-dimensional nanomaterials.

Another important application of in situ manipulation techniques is about nanoassembly with high degree of automation, which could speed up the fabrication of the nanodevices in the future. C. Zhou et al. proposed an automated axis alignment method for a nanomanipulator inside the SEM by recognizing the position of a closed-loop controlled end-effector. Over these years, carbon nanotube (CNT) was proved to have potential applications in the integration of large-scale interconnections, a key component in the manufacturing of nanodevices. A method of multiwalled carbon nanotubes (MWCNTs) fusion by electronic beam irradiation inside SEM was reported and proved reliable by D. Shen and coworkers.

So far there are less reports on the applications of in situ SEM techniques for biomedical research because of the challenges in high vacuum operating environment and complicated bio-sample preparation steps. Here, M. A. Rad et al. performed in situ local direct observation and manipulation of a biological sample by controlling the environmental conditions, demonstrating the ability to observe spheroplast cells under electron microscope without the need of sample coating for the first time, which could open great opportunities for in situ SEM-aid biomedical research.

Overall, the objectives of the special issue have been reached in terms of advancing the current state of the art of in situ SEM nanomanipulation and nanomechanical/electrical characterization techniques. Several basic problems in these areas were well addressed and most of the proposed contributions exhibited very promising results that outperform existing studies in the community. Some results were even firstly reported in these areas, such as in situ torsion testing of microwires.

